# Supplementary material for: Tumor-associated hematopoietic stem and progenitor cells positively linked to glioblastoma progression
Source: Nat Commun. 2021 Jun 23;12:3895. doi: 10.1038/s41467-021-23995-z (PMC8222381; doi:10.1038/s41467-021-23995-z)
Supplement: Supplementary file 1 — Supplementary Information [file 41467_2021_23995_MOESM1_ESM.pdf]

SUPPLEMENTARY INFORMATION

Tumor-associated hematopoietic stem and progenitor cells positively linked to glioblastoma progression.

I-Na Lu, Celia Dobersalske *et al.*

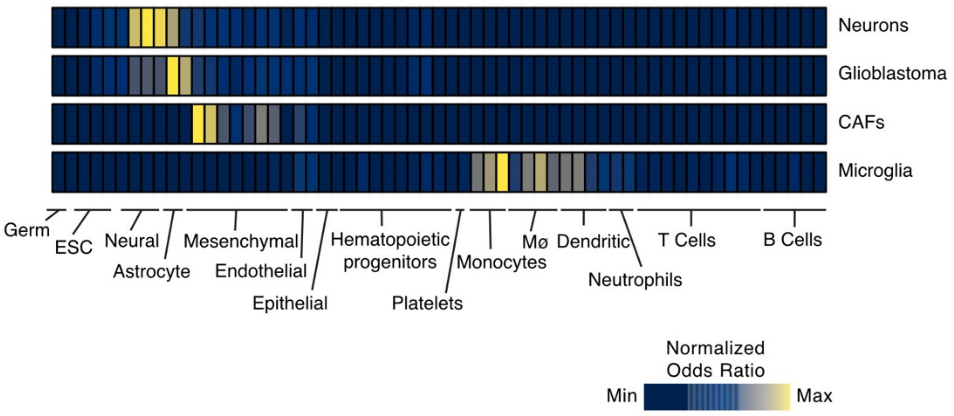

Supplementary Fig. 1: Additional cell types tested by Syllogist. Each colored box represents a normalized odds ratio ranging from 0 (blue) to 1 (yellow). Identifiers for publicly available datasets used in this analysis are reported in Supplementary Data 10. CAFs, Cancer Associated Fibroblasts; ESC, Embryonic Stem Cell, Mø, Macrophages.

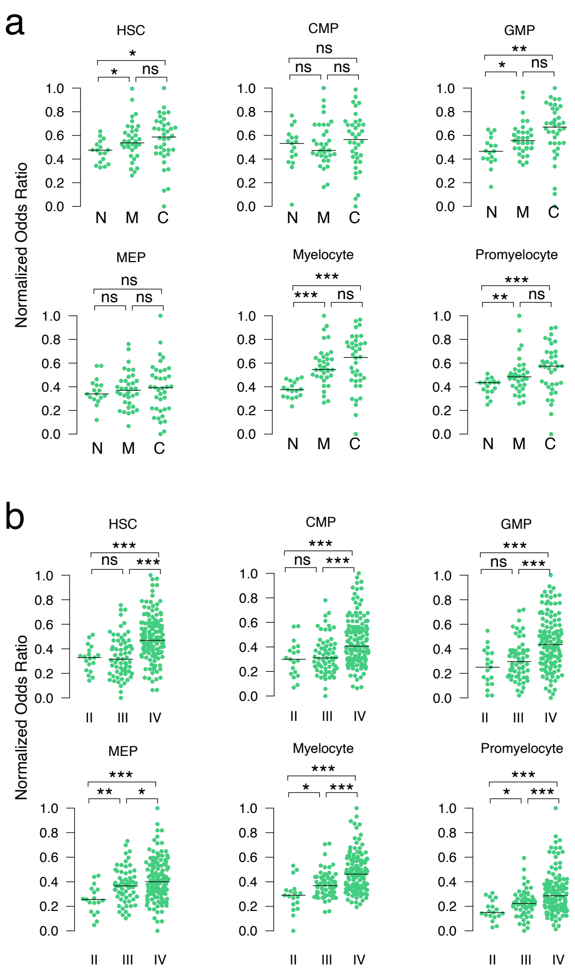

Supplementary Fig. 2: a Association of six HSPC subsets with brain tumor locations from normal brains (N, *n* = 17 samples), glioblastoma margins (M, *n* = 36) and cores (C, *n* = 38). **b** Association of six HSPC subsets with diffuse astrocytomas (WHO grade II, *n* =19 samples), anaplastic astrocytoma (WHO grade **III**, *n* = 67) and glioblastoma (WHO grade IV, *n* = *143*). Dot plots represent normalized odds ratios computed by Syllogist. Line across dot plots represent median values for each variable.For **a** and **b**, *p* values were determined by 2-tailed, unpaired Student's *t*-test with correction by the Benjamini-Hochberg procedure. *, $p < 0.05$; **, $p < 0.01$; ***, $p < 0.001$, ns, not significant. Exact *p* values are reported in Supplementary Data 2 and 3, respectively Source data are provided as a Source Data file. HSC, Hematopoietic Stem Cell; CMP, Common Myeloid Progenitor; GMP Granulocyte-Monocyte Progenitor; MEP, Megakaryocyte-Erythroid Progenitor.

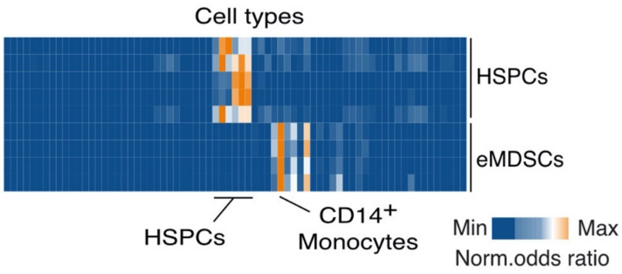

Supplementary Fig. 3: Syllogist transcriptome analysis of eMDSCs and HSPCs indicating distinct signals without overlap. Data were quantile normalized before analysis. Information about the publicly available datasets used in this analysis are reported in Supplementary Data 10.

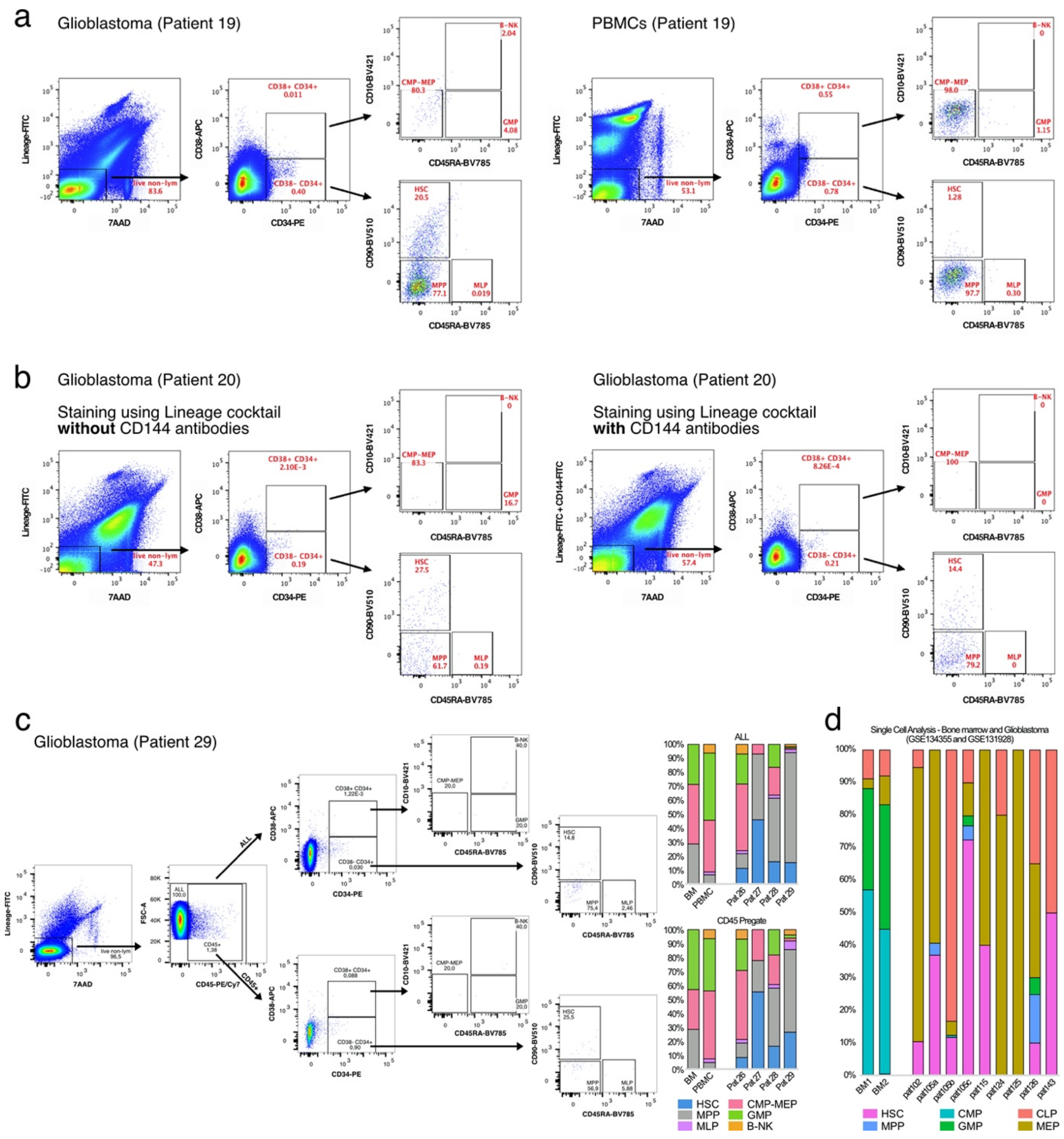

Supplementary Fig. 4: Flow cytometric analyses of circulating and tissue-associated HSPCs. **a** HSPCs subsets as shown in Fig. 3b were measured from samples of a glioblastoma single cell suspension (left panel) and PBMCs (right panel) from the same patient. Note the HSC gate in both samples indicating enrichment in glioblastoma compared to blood. **b** Glioblastoma cell suspensions were stained with a lineage marker cocktail with and without anti-CD144 antibodies (*n* = 1 patient). **c** Representative flow cytometry profile of glioblastoma tissue gated for HSPC subsets with and without CD45 pre-gating. Stacked barplot indicates proportions of HSPC subsets in PBMC (*n* = 1), BM (*n* = 1) and glioblastoma samples (*n* = 4) with and without

CD45 pre-gating. **d** Annotation of HSPC subsets in publicly available scRNA-Seq data of 9 glioblastoma samples (*n* = 7 patients) and 2 bone marrow samples. Source data of c and d are provided as a Source Data file. HSC, Hematopoietic Stem Cell; MPP, Multipotent Progenitor; MLP, Multi-Lymphoid Progenitor; CMP-MEP, Common Myeloid Progenitor and Megakaryocyte-Erythroid Progenitor; GMP, Granulocyte-Monocyte Progenitor; B-NK, B-NK Progenitor

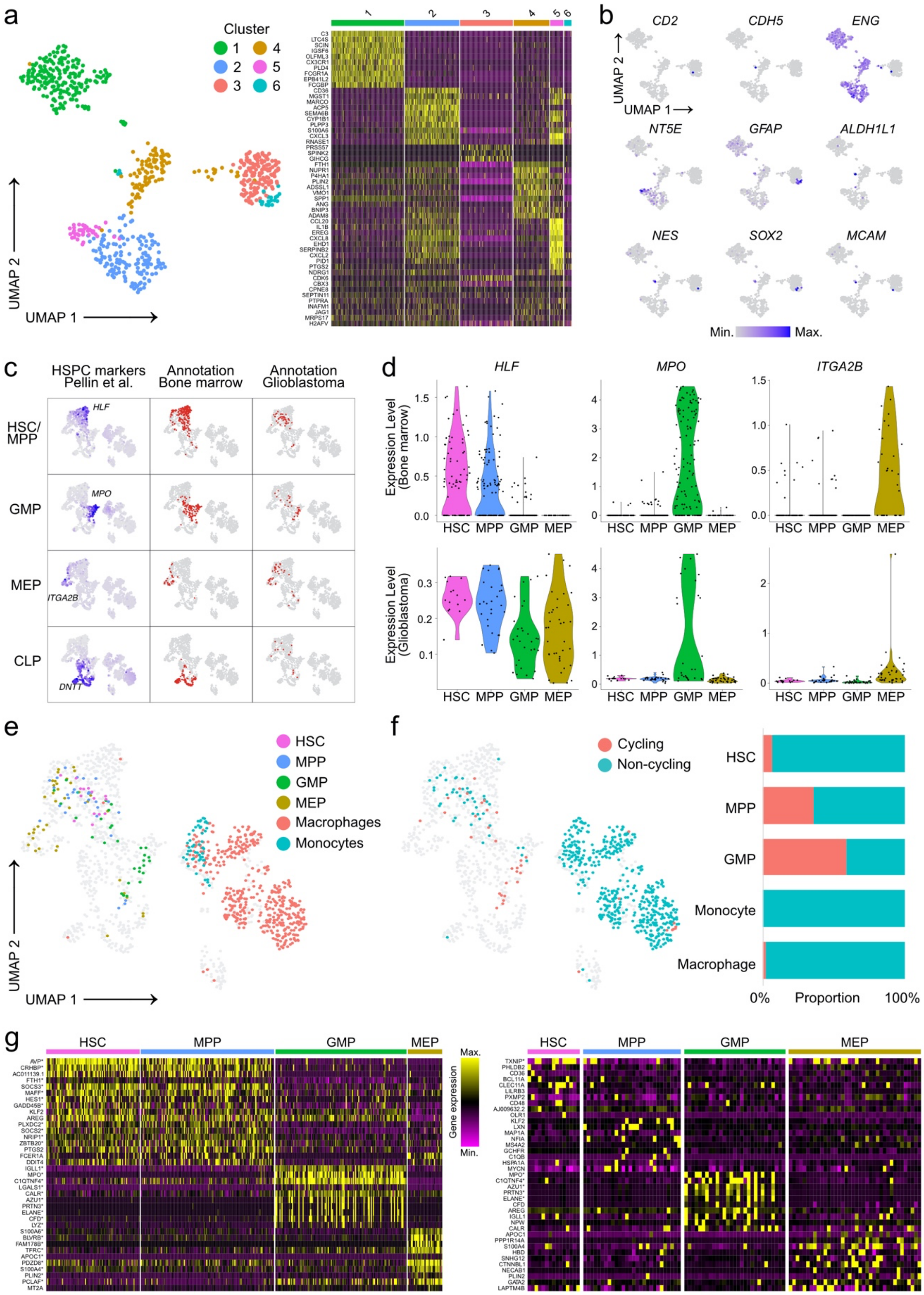

Supplementary Fig. 5: Comparison of tumor-associated HSPCs with canonical hematopoietic progenitors by scRNA-Seq

a Heatmap of differentially expressed genes between graph-based clusters in the CD45+CD34+-enriched glioblastoma dataset. **b** Marker expression for lymphoid

(*CD2*), endothelial (*CDH5, MCAM*), mesenchymal (*ENG, NT5E*), astrocytic (*GFAP, ALDH1L1*) and neuronal cells (*NES, SOX2*) of the glioblastoma dataset. **c** UMAP plots highlighting the expression of markers specific for the hematopoietic stem and progenitor cell (HSPC) subsets HSC/MPP (*HLF*), GMP (*MPO*), MEP (*ITGA2B*) and CLP (*DNTT*) in the integrated dataset (first column), and cells annotated by SingleR in the bone marrow (second column) and glioblastoma (third column) dataset. HSC, Hematopoietic Stem Cell; MPP, Multipotent Progenitor; GMP, Granulocyte-Monocyte Progenitor; MEP, Megakaryocyte-Erythroid Progenitor; CLP, Common Lymphoid Progenitor. **d** Violin plots showing selected marker expression from c in the bone marrow and glioblastoma HSPC subsets annotated by SingleR. **e** UMAP plot of the integrated dataset highlighting glioblastoma-annotated HSPCs by subset. **f** Cycling and non-cycling cells computed by Seurat for the glioblastoma HSPCs (left) and stacked barplots showing the respective cycling and non-cycling proportions for the indicated cell types (right). **g** Heatmaps showing the top overexpressed genes between HSPC subsets from the bone marrow (left) and the glioblastoma (right) dataset. Asterisks show genes significantly regulated after adjustment ($p < 0.05$, two-tailed Student's t-test with Bonferroni correction). Source data are provided as a Source Data file.

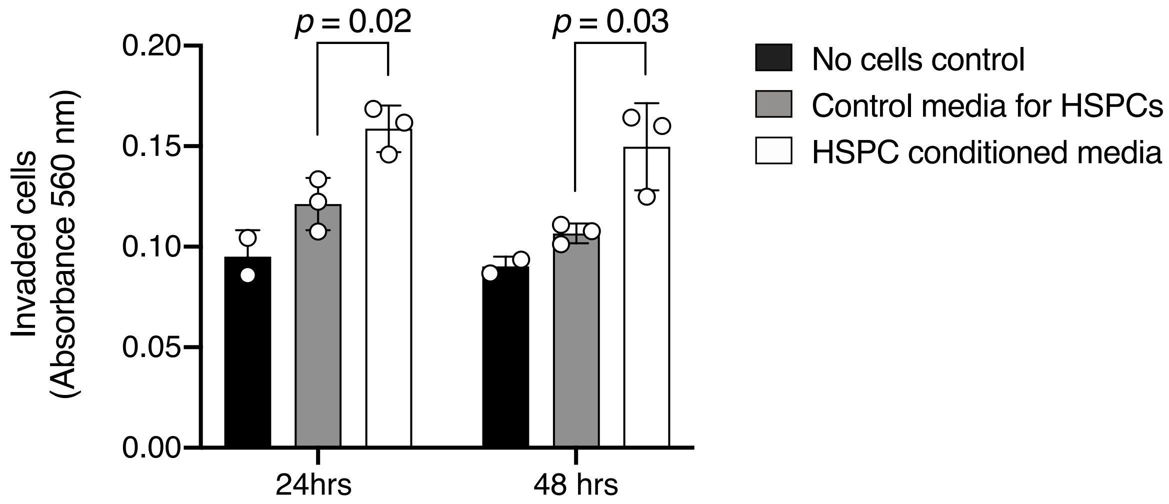

Supplementary Fig. 6: Invasion assay using hippocampus-derived adult human neural progenitor cells (AHNP) and control or hematopoietic stem and progenitor cell (HSPC) conditioned media. Invaded cells were stained and quantified by colorimetric measurement as described in the manufacturer's protocol. Results are presented as mean ± standard deviation. Statistics derived from n = 3 technical replicates from a representative experiment of 3. p values determined by unpaired two-tailed Student's t-test. Source data are provided as a Source Data file.

a

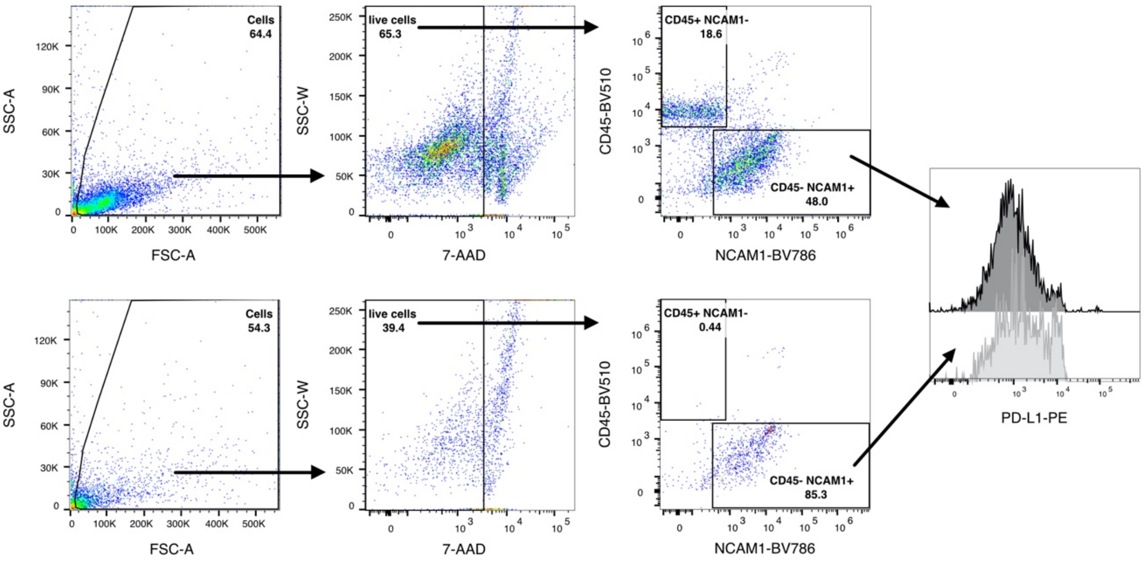

b

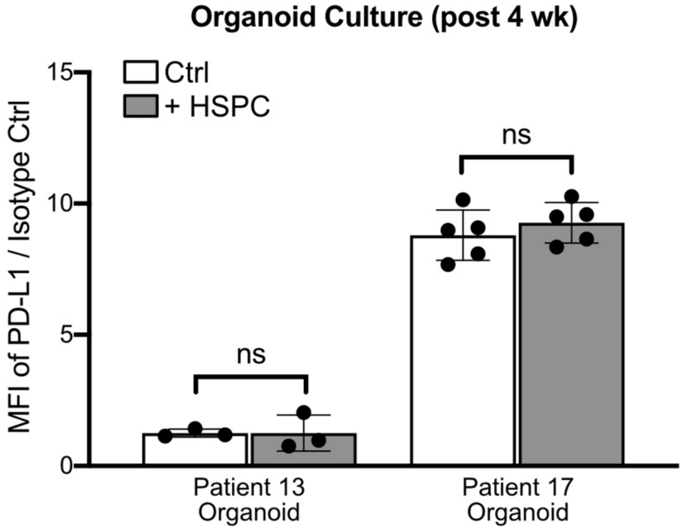

Supplementary Fig. 7: Co-culture of hematopoietic stem and progenitor cells (HSPCs) within organoids does not lead to PD-L1 upregulation. **a** Representative flow cytometry profiles of organoids with stainings and gatings used to distinguish HSPCs (7-AAD$^-$CD45$^+$NCAM1$^-$) from tumor cells (7-AAD$^-$CD45$^-$NCAM1$^+$). **b** Bar plot represents PD-L1 normalized mean fluorescence intensity (MFI) of tumor cells derived from organoids culture in the presence and absence of HSPC for two patients (patient 13, n = 3 technical replicates, patient 17, n = 5). Results are presented as mean ± standard deviation. ns, not significant, two-tailed, unpaired Student’s t-test. Source data of b are provided as a Source Data file.

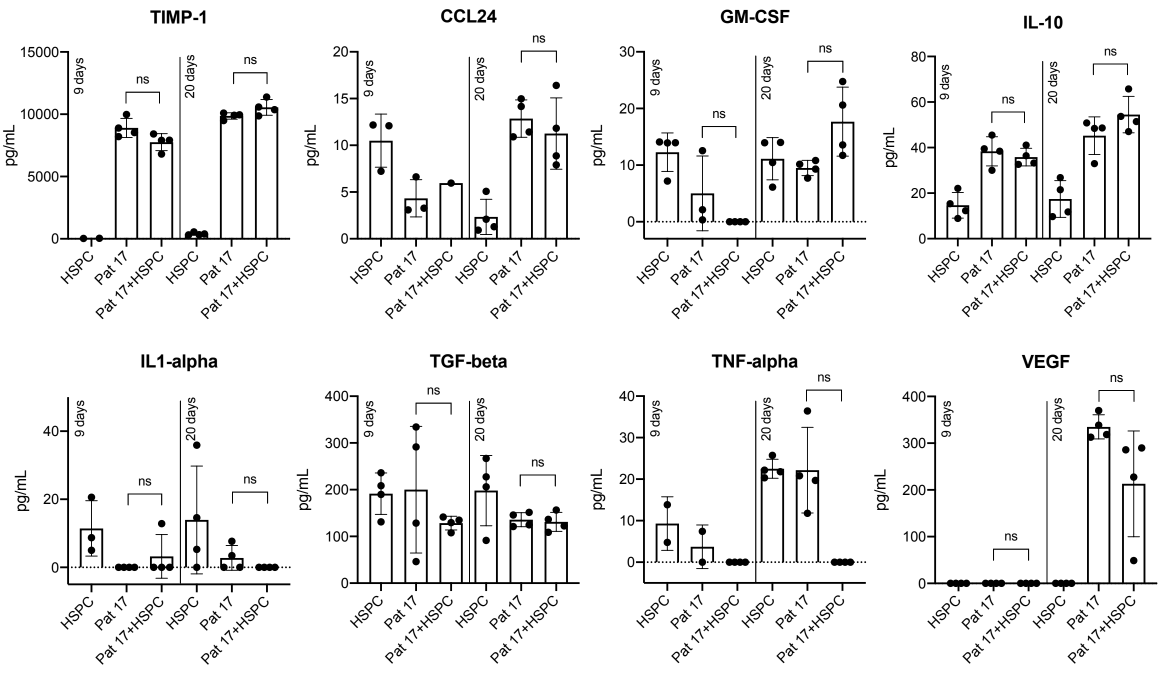

Supplementary Fig. 8: Cytokine ELISA array from conditioned media of co-cultured organoids from patient-derived glioblastoma cells (patient 17) in the presence or absence of hematopoietic stem and progenitor cells (HSPCs). Conditioned media was collected at day 9 and day 20 of co-culture. n = 1–4 technical replicates from one representative experiment of 2. Data are presented as mean ± standard deviation. p values were determined using two-tailed, unpaired Student's t-test corrected with the Benjamini-Hochberg procedure. Source data are provided as a Source Data file. TIMP-1, TIMP metallopeptidase inhibitor 1; CCL24, CC-chemokine ligand 24; GM-CSF, Granulocyte-Macrophage Colony-Stimulating Factor; IL-10, Interleukin-10; IL1-alpha, Interleukin 1 alpha; TGF-beta, Transforming Growth Factor Beta; TNF-alpha, Tumor Necrosis Factor; VEGF, Vascular Endothelial Growth Factor.

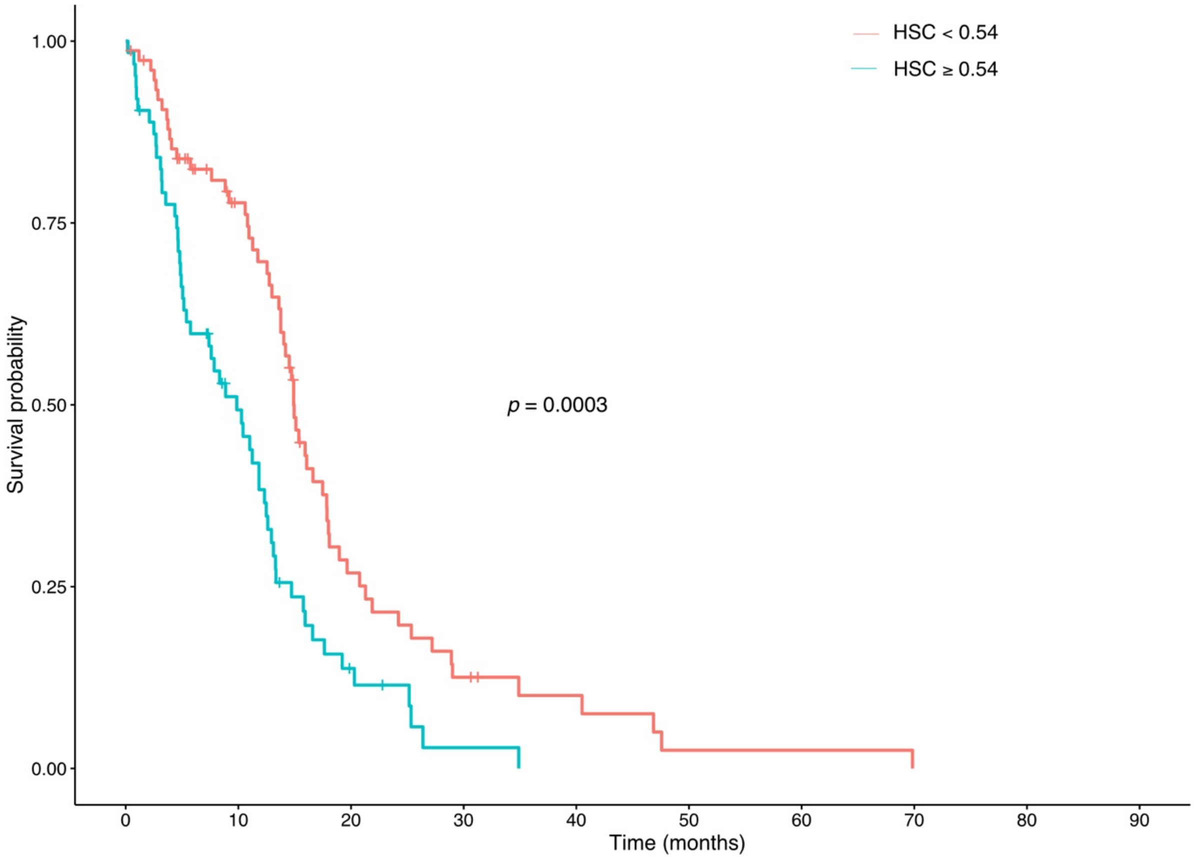

Supplementary Fig. 9: Kaplan-Meier plot of *IDH* wildtype glioblastoma for HSC^{high} (*n* = 63) and HSC^{low} (*n* = 76) patients using data reported in Figure 6. Two-tailed logrank test. HSC, Hematopoietic Stem Cell.

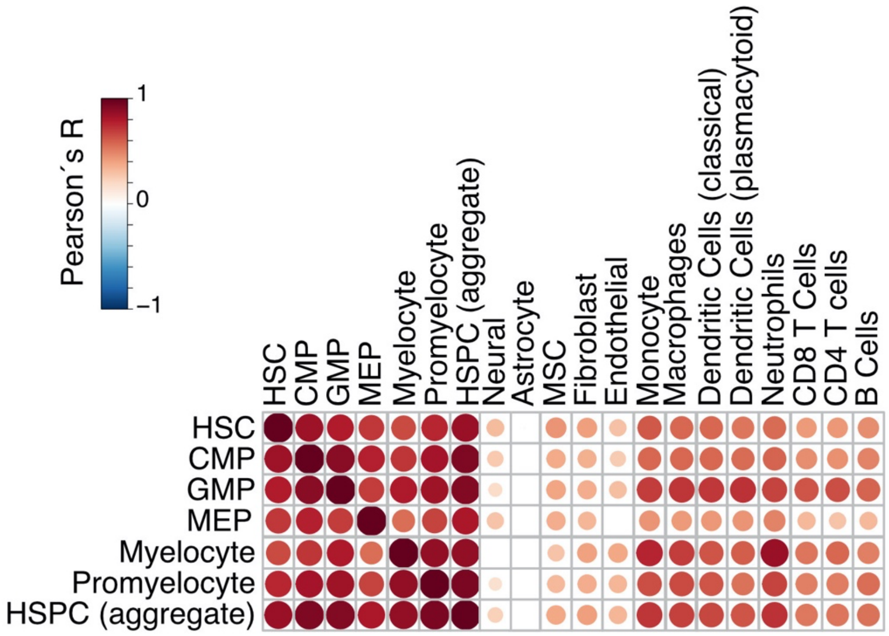

Supplementary Fig. 10: Correlation matrix of HSPC and mature immune cell subsets signals computed by Syllogist on the GBM TCGA dataset. Pearson correlation coefficients with significant values (p < 0.05, two-tailed Student's t-test) are shown as circles, with circle size and color matching Pearson correlation coefficients from -1 (blue) to 1 (dark red). Empty squares represent correlation coefficients with $p \geq 0.05$). HSC, Hematopoietic Stem Cell; CMP, Common Myeloid Progenitor; GMP, Granulocyte-Monocyte Progenitor; MEP, Megakaryocyte-Erythroid Progenitor; HSPC, Hematopoietic Stem and Progenitor Cell; MSC, Mesenchymal Stem Cell.

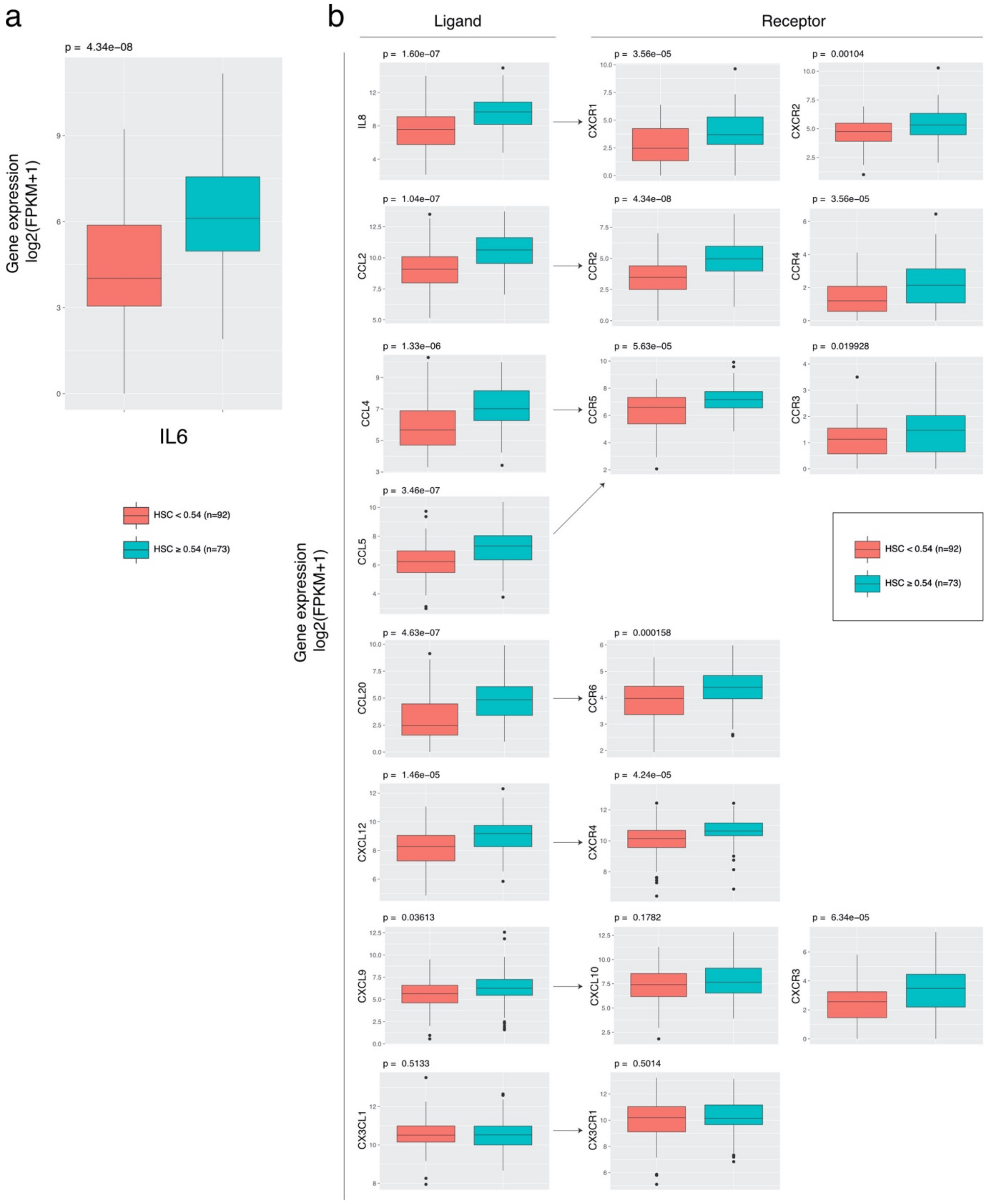

Supplementary Fig. 11: a Gene expression of IL-6 in HSC^{high} (n = 73) and HSC^{low} (n = 92) patient samples. **b** Expression of different chemokine ligands and the respective receptors in HSC^{high} (n = 73) and HSC^{low} (n = 92) patient samples. In **a** and **b**, boxplots are drawn with boxes representing the interquartile range (IQR), a line across the box indicating the median, and whiskers indicating 1.5 × IQR. Outliers are shown as closed dots. p values are determined using a two-tailed Wilcoxon-

Mann-Whitney U test corrected with the Benjamini-Hochberg procedure. HSC, Hematopoietic Stem Cell.
