# Supplementary material for: Tumor-associated hematopoietic stem and progenitor cells positively linked to glioblastoma progression
Source: Nat Commun. 2021 Jun 23;12:3895. doi: 10.1038/s41467-021-23995-z (PMC8222381; doi:10.1038/s41467-021-23995-z)
Supplement: Supplementary file 3 — Description of Additional Supplementary Files [file 41467_2021_23995_MOESM3_ESM.pdf]

Description of Additional Supplementary Files

Supplementary Data 1: Top 80 gene signatures used as reference by Syllogist listed in order of decreasing specificity index S

Supplementary Data 2: Cell type estimation results from the GSE59612 dataset [https://www.ncbi.nlm.nih.gov/geo/query/acc.cgi?acc=GSE59612]. *p* values determined by 2-tailed, unpaired Student's t-test with correction by the Benjamini-Hochberg procedure

Supplementary Data 3: Cell type estimation results from LGG-GBM dataset. *p* values determined by 2-tailed, unpaired Student's t-test with correction by the Benjamini-Hochberg procedure

Supplementary Data 4: Information on patients recruited for the study with reference to experiments performed. KPS, Karnofsky Performance Score; *MGMT*, O6-methylguanine-DNA methyltransferase; *ATRX*, ATP-dependent helicase ATRX; Wt, wild type; Mut, mutant; Met, methylated; Unmet, unmethylated

Supplementary Data 5: Differentially expressed genes of the scRNA HSC subset. *p* values determined by 2-tailed, unpaired Student's *t*-test with Bonferroni correction (Seurat default)

Supplementary Data 6: Differentially expressed genes of the scRNA MPP subset. *p* values determined by 2-tailed, unpaired Student's *t*-test with Bonferroni correction (Seurat default)

Supplementary Data 7: Differentially expressed genes of the scRNA GMP subset. *p* values determined by 2-tailed, unpaired Student's *t*-test with Bonferroni correction (Seurat default)

Supplementary Data 8: Differentially expressed genes of the scRNA MEP subset. *p* values determined by 2-tailed, unpaired Student's *t*-test with Bonferroni correction (Seurat default)

Supplementary Data 9: Univariate Cox regression analysis of selected variables. Two-tailed likelihood ratio test corrected by the Benjamini-Hochberg procedure

Supplementary Data 10: Publicly available datasets analyzed in this study.
