# Supplementary material for: Tumor-associated hematopoietic stem and progenitor cells positively linked to glioblastoma progression
Source: Nat Commun. 2021 Jun 23;12:3895. doi: 10.1038/s41467-021-23995-z (PMC8222381; doi:10.1038/s41467-021-23995-z)
Supplement: Supplementary file 5 — Reporting summary [file 41467_2021_23995_MOESM5_ESM.pdf]

nature research

Corresponding author(s): Igor Cima
Last updated by author(s): May 25, 2021

Reporting Summary

Nature Research wishes to improve the reproducibility of the work that we publish. This form provides structure for consistency and transparency in reporting. For further information on Nature Research policies, see Authors & Referees and the Editorial Policy Checklist .

Statistics

For all statistical analyses, confirm that the following items are present in the figure legend, table legend, main text, or Methods section.

n/a	Confirmed	
☐	☒	The exact sample size (*n*) for each experimental group/condition, given as a discrete number and unit of measurement
☐	☒	A statement on whether measurements were taken from distinct samples or whether the same sample was measured repeatedly
☐	☒	The statistical test(s) used AND whether they are one- or two-sided<br>*Only common tests should be described solely by name; describe more complex techniques in the Methods section.*
☐	☒	A description of all covariates tested
☐	☒	A description of any assumptions or corrections, such as tests of normality and adjustment for multiple comparisons
☐	☒	A full description of the statistical parameters including central tendency (e.g. means) or other basic estimates (e.g. regression coefficient) AND variation (e.g. standard deviation) or associated estimates of uncertainty (e.g. confidence intervals)
☐	☒	For null hypothesis testing, the test statistic (e.g. *F*, *t*, *r*) with confidence intervals, effect sizes, degrees of freedom and *P* value noted<br>*Give P values as exact values whenever suitable.*
☒	☐	For Bayesian analysis, information on the choice of priors and Markov chain Monte Carlo settings
☒	☐	For hierarchical and complex designs, identification of the appropriate level for tests and full reporting of outcomes
☐	☒	Estimates of effect sizes (e.g. Cohen's *d*, Pearson's *r*), indicating how they were calculated

Our web collection on statistics for biologists contains articles on many of the points above.

Software and code

Policy information about availability of computer code

Data collection	FACS Diva BD Biosciences v 8.0.1.1 ; Zeiss ZEN 2.3 Imaging Software
Data analysis	Bioinformatic and statistical data analysis was performed in the R environment, version 3.6.1. Some statistical analyses were performed using Prism software (v 8.4, GraphPad). Image data were analyzed using ZEISS ZEN 2.3 Imaging Software. Multiplex ELISA was analzyed using ImageJ Software 1.46r. Illumina basecall (.bcl) data were converted and demultiplexed to FASTQ files using the bcl2fastq v2.20 software. RNA-Sequencing results were analyzed using the 10x Genomics Cell Ranger 4.0.0 pipeline and Seurat 4.0.

For manuscripts utilizing custom algorithms or software that are central to the research but not yet described in published literature, software must be made available to editors/reviewers. We strongly encourage code deposition in a community repository (e.g. GitHub). See the Nature Research guidelines for submitting code & software for further information.

Data

Policy information about availability of data

All manuscripts must include a data availability statement. This statement should provide the following information, where applicable:
- Accession codes, unique identifiers, or web links for publicly available datasets
- A list of figures that have associated raw data
- A description of any restrictions on data availability

References to repositories for publicly available RNA-Seq datasets analyzed during this study are listed in Supplementary Table 10. Single cell RNA-Seq data generated in this study (Fig. 4 and Supplementary Fig. 5) are available at the Gene Expression Omnibus under the accession number GSE165238. The source data underlying Figures 1–6 and Supplementary Figures 2 and 4–8 are provided as a Source Data file. All the other data supporting the findings of this study are available within the article and its supplementary information files and from the corresponding author upon reasonable request.

Field-specific reporting

Please select the one below that is the best fit for your research. If you are not sure, read the appropriate sections before making your selection.

☒ Life sciences ☐ Behavioural & social sciences ☐ Ecological, evolutionary & environmental sciences

For a reference copy of the document with all sections, see nature.com/documents/nr-reporting-summary-flat.pdf

Life sciences study design

All studies must disclose on these points even when the disclosure is negative.

Sample size	Sample sizes were determined based on preliminary experiments in the case of in vitro and organoid experiments (Figure 5) and were considered appropriate based on the consistency of the observed effects between experiments. For patient-derived samples, we were limited by tissue availability, and sample size was considered adequate based on comparisons with data reported from other studies in the field that used similar sample sizes (e.g. Neftel et al. Cell 178,835, analyzed in supplementary Figure 4d).
Data exclusions	Organoid culture from one patient sample was excluded in the experiments shown in Figure 5f–5j and Supplementary Figure 7 as we did not achieve sustained growth for this particular sample. One glioblastoma patient sample was excluded from scRNA-Seq because of poor quality of cell suspension (>20% cell death as measured by trypan blue exclusion)
Replication	- Flow cytometry analysis of HSPC subsets was performed on 7 GBM patients and 5 non-GBM patients.<br>- CFC assay was performed on 8 GBM patients and 6 non-GBM patients.<br>- CXCL12 IHC staining was performed on 7 GBM patients.<br>- Immunofluorescence studies were performed on 4 GBM patients and 4 autoptic tissues.<br>- All in vitro experiments involving cell lines were repeated for a minimum of three times.<br>- Organoid cultures were performed twice, each time with 2 different patient samples, using 3 to 8 technical replicates for each condition<br>- Multiplex ELISA was performed on 2 organoids experiments using 2 different patient samples. Each cytokine was measured using 4 technical replicates for each condition.<br>-Single cell RNA-Seq was performed on 1 glioblastoma sample, 1 tumor-free sample, 1 bone marrow sample and 1 blood sample.
Randomization	Not relevant - no treatment groups.
Blinding	Not relevant - no treatment groups.

Reporting for specific materials, systems and methods

We require information from authors about some types of materials, experimental systems and methods used in many studies. Here, indicate whether each material, system or method listed is relevant to your study. If you are not sure if a list item applies to your research, read the appropriate section before selecting a response.

Materials & experimental systems

n/a	Involved in the study	
☐	☒	Antibodies
☐	☒	Eukaryotic cell lines
☒	☐	Palaeontology
☒	☐	Animals and other organisms
☐	☒	Human research participants
☒	☐	Clinical data

Methods

n/a	Involved in the study	
☒	☐	ChIP-seq
☐	☒	Flow cytometry
☒	☐	MRI-based neuroimaging

Antibodies

Antibodies used

Antibody;Clone;Company;Catalogue number;Dilution
BV421-CD10; HI10a; BD Biosciences; 562902; 1:50
BV510-CD90; 5E10; BD Biosciences; 563070; 1:50
BV711-CD135; 4G8; BD Biosciences; 563908; 1:50
BV785-CD45RA; HI100; BD Biosciences; 563870; 1:50
PE-CD34; 581; BD Biosciences; 560941; 1:25
Lineage Cocktail 1; 3G8L27MφP9, NCAM16.2, SJ25C1, SK7; BD Biosciences; 340546; 1:25
APC-CD38; HIT2; eBioscience; 17-0389-41; 1:50
PE-PD-L1; 29E.2A3; BioLegend; 329705; 1:100
FITC-CD144; 55-7H1; BD Biosciences;560874 ;1:25
CXCL12; polyclonal; Abcam; ab9797; 1:600

	CD34; QBEND/10; Leica; NCL-L-END; 1:250<br>CD45; polyclonal; Abcam; ab10559; 1:250<br>CD45-PE/Cyanine7; 2D1; BioLegend, 368531; 1:50<br>BV510-CD45; 2D1; BioLegend; 368525; 1:20<br>BV786-CD56; NCAM16.2; BD Biosciences; 564058; 1:20<br>anti-rabbit Alexa 488; polyclonal; Invitrogen; A11001; 1:800<br>anti-mouse Alexa 555; polyclonal; Invitrogen; A21422; 1:800
Validation	Antigen;Clone;Company; Link; Reference<br>CD10; HI10a; BD Biosciences; https://www.bdbiosciences.com/ds/pm/tds/562902.pdf; doi: 10.1084/jem.181.6.2271<br>CD90; 5E10; BD Biosciences; https://www.bdbiosciences.com/ds/pm/tds/563070.pdf; doi: 10.1186/1479-5876-11-28<br>CD135; 4G8; BD Biosciences; https://www.bdbiosciences.com/ds/pm/tds/563908.pdf; ISBN: 0815327455 9780815327455<br>CD45RA; HI100; BD Biosciences; https://www.bdbiosciences.com/ds/pm/tds/563870.pdf; doi: 10.4049/jimmunol.0901967<br>CD34; 581; BD Biosciences; https://www.bdbiosciences.com/ds/pm/tds/560941.pdf; PMID: 7680152<br>3G8L27MϕP9, NCAM16.2, SJ25C1, SK7; BD Biosciences; https://www.bdbiosciences.com/ds/is/tds/23-3546.pdf<br>CD38; HIT2; eBioscience; https://www.thermofisher.com/order/genome-database/generatePdf?productName=CD38&assayType=PRANT&detailed=true&productId=17-0389-41; doi: 10.1189/jlb.0307153<br>PD-L1; 29E.2A3; BioLegend; https://www.biolegend.com/en-us/products/pe-anti-human-cd274-b7-h1-pd-l1-antibody-4375; DOI: https://doi.org/10.4049/jimmunol.170.3.1257<br>CD144; 55-7H1; BD Biosciences; https://www.bdbiosciences.com/ds/pm/tds/560874.pdf<br>CXCL12; polyclonal; Abcam; https://www.abcam.com/sdf1-antibody-ab9797.html<br>CD34; QBEND/10; Leica; DOI: 10.1111/j.1365-2559.1990.tb00713.x<br>CD45; polyclonal; Abcam; PMID: 29123364<br>CD45; 2D1; BioLegend, https://www.biolegend.com/en-us/products/pe-cyanine7-anti-human-cd45-antibody-14721; doi: 10.1093/intimm/dxh112<br>CD56; NCAM16.2; BD Biosciences; 564058; https://www.bdbiosciences.com/ds/pm/tds/564058.pdf; ISBN-13 : 978-0192623768

Eukaryotic cell lines

Policy information about cell lines

Cell line source(s)	The cell lines used in this study were purchased from ATCC with catalog numbers: ATCC CRL-1690 (T98-G) , ATCC HTB-14 (U-87), ATCC CRL-2611 (LN-229)
Authentication	No authentication was performed.
Mycoplasma contamination	Mycoplasma contamination is routinely assessed in our lab. No mycoplasma contamination was recorded during experiments reported in manuscript.
Commonly misidentified lines (See ICLAC register)	No commonly misidentified line was used according to ICLAC register version 10.

Human research participants

Policy information about studies involving human research participants

Population characteristics	Population characteristics are outlined in Supplementary Table 4.
Recruitment	Tissue samples (n = 27) were obtained from donors admitted for surgery at the Department of Neurosurgery at the University Hospital Essen after acquiring informed consent. To avoid potential biases, comparisons of samples from human research participants (Figure 3 and supplementary Figure 4c) were conducted by recruiting unselected, consecutive patients derived from a single source and processed identically. Two additional patient samples were obtained from the University of Bonn and were used to generate cell cultures for the organoid experiments shown in Figures 5f-5j.
Ethics oversight	Biosamples were obtained from 29 patients after informed consent at the Departments of Neurosurgery of the University Hospitals Bonn and Essen. At each site, the local ethics committees approved the study (University Bonn #182/08; University of Duisburg-Essen, #19_8706_BO). Human biological samples and related data collected in Essen were provided by the Westdeutsche Biobank Essen (WBE, University Hospital Essen, University of Duisburg-Essen, Germany, approval 19_WBE_074).

Note that full information on the approval of the study protocol must also be provided in the manuscript.

Flow Cytometry

Plots

Confirm that:

- [x] The axis labels state the marker and fluorochrome used (e.g. CD4-FITC).
- [x] The axis scales are clearly visible. Include numbers along axes only for bottom left plot of group (a 'group' is an analysis of identical markers).
- [x] All plots are contour plots with outliers or pseudocolor plots.
- [x] A numerical value for number of cells or percentage (with statistics) is provided.

Methodology

Sample preparation	Fresh surgical tissue was placed in ice-cold DMEM/F12-based transport medium in the operating room and received on ice at the lab within 30 minutes thereafter. The tumor tissues were subsequently cut into small pieces and homogenized in Iscove's Modified Dulbecco's Medium (IMDM) with 0.11 DMC U/mL neutral protease (Nordmark Biochemicals) at 37°C for 1-2 hour in a shaker-incubator (shaking speed: 250 rpm). The homogenized tissues were centrifuged for 10 minutes at 300 ×g, resuspended in IMDM and filtered through a 40 µm cell strainer for the following FACS analysis.
Instrument	All flow cytometry analyses were performed using BD FACSCelesta™ Flow Cytometer with BVYG Configuration (no. 660345).
Software	FACS Diva BD Biosciences v 8.0.1.1; FlowJo (Version 10.6.2)
Cell population abundance	No cell-sorting performed.
Gating strategy	Gating strategies are provided in Fig. 3c, Fig. 5a and Supplementary Fig. 4a-c and Supplementary Fig.7

- [x] Tick this box to confirm that a figure exemplifying the gating strategy is provided in the Supplementary Information.
